# Detecting differences in the topology of scale-free networks grown under time-dynamic topological fitness

**DOI:** 10.1038/s41598-020-67156-6

**Published:** 2020-06-30

**Authors:** Dimitrios Tsiotas

**Affiliations:** 1 Department of Regional and Economic Development, Agricultural University of Athens, Greece, Nea Poli, Amfissa 33100 Greece; 2 Department of Planning and Regional Development, University of Thessaly, Pedion Areos, Volos 38334 Greece; 3 Laboratory of Complex Systems, Department of Physics, Faculty of Sciences, International Hellenic University, Kavala Campus, St. Loukas, 65404 Greece

**Keywords:** Complex networks, Statistical physics

## Abstract

The fitness model was introduced in the literature to expand the Barabasi-Albert model’s generative mechanism, which produces scale-free networks under the control of degree. However, the fitness model has not yet been studied in a comprehensive context because most models are built on invariant fitness as the network grows and time-dynamics mainly concern new nodes joining the network. This mainly static consideration restricts fitness in generating scale-free networks only when the underlying fitness distribution is power-law, a fact which makes the hybrid fitness models based on degree-driven preferential attachment to remain the most attractive models in the literature. This paper advances the time-dynamic conceptualization of fitness, by studying scale-free networks generated under topological fitness that changes as the network grows, where the fitness is controlled by degree, clustering coefficient, betweenness, closeness, and eigenvector centrality. The analysis shows that growth under time-dynamic topological fitness is indifferent to the underlying fitness distribution and that different topological fitness generates networks of different topological attributes, ranging from a mesh-like to a superstar-like pattern. The results also show that networks grown under the control of betweenness centrality outperform the other networks in scale-freeness and the majority of the other topological attributes. Overall, this paper contributes to broadening the conceptualization of fitness to a more time-dynamic context.

## Introduction

The scale-free (SF) property^1,2^ describes networks where a few nodes undertake the major load of connectivity and it is generally related to the hierarchical structure that real-world communication-systems develop to deal with complexity^3,4^. Although SF networks are claimed to be rare in nature^5^, empirical research^2,3,6,7^ has shown that are of great importance in the real-world because they are related to biological (e.g. protein), economic (e.g. interbank, airline), technological (e.g. the internet, the World Wide Web), and social (e.g. collaboration and citation) applications^2,3,6,7^. Also, SF networks abound in scientific literature and they are a common null (i.e. reference) model in related research^8^. These reasons make the SF property a major concept in network science^1,2,9^. In terms of definition, a network is deemed SF when its degree distribution *p*(*k*) follows a power-law (PL) distribution *p*(*k*) ~ *k*^–*γ*^^1^, where *k* is the node degree and *γ* > 1, so that the Riemann zeta function will be finite^10^. Empirical research has shown that SF networks usually have their PL exponent (*γ*) ranging within the interval 2<*γ* < 3^1^, although this is not a defining condition and these typical bounds may vary^11^. The most common generative model of SF networks is the *Barabasi-Albert* (BA) model^2^, which is based on the preferential attachment (PA) growth process^1,12^ and defines that the probability (*p*_*i*_) for a node *i* to gain a connection is proportional to its degree *k*_*i*_^2,13^, according to the relation:

1 $${p}_{i}={k}_{i}/\mathop{\sum }\limits_{j=1}^{n}{k}_{j}.$$

where *n* is the total number of nodes in the network.

In the BA model, first-comers (i.e. first nodes joining the network) are more likely to become hubs (i.e. highly connected nodes), whereas newcomers prefer (whence the term preferential comes from) to connect with hubs and to benefit from their high connectivity^8,13^. The PA process leads to the emergence of hierarchies in the network, where hubs undertake the major load of connectivity and preserve their privilege at future network growth^8^. This is reflected in the PL shape of the degree distribution expressing an abrupt declining frequency of nodes of higher degree^1,2^. In epistemological terms, the PA process is based on the stochastic *Yule process*, which was introduced by the British statistician George Udny Yule^14^ during the study of the evolution of species. In sociology, an aspect of PA can be found back to the *Matthew effect*^15^ expressed by the *rich get richer and the poor get poorer* motto, and in economics, the PA is related to the *Gibrat’s law*^16^ describing the proportional growth of firms in terms of their absolute size. However, in real-world applications, more factors than age and the number of existing connections seem to influence the ability of nodes to increase their connectivity^13^. To explain empirical observations of latecomers who can also become hubs^9^, such as the case of Google in the World Wide Web^13^, the authors of^12^ introduced a variant of the BA model taking into account an intrinsic attractiveness of nodes. This attractiveness is called *node fitness* and is expressed by the (non-negative) weights *φ*_*i*_ applied to the node degrees *k*_*i*_ that are configuring the connecting probability as follows:

2 $${p}_{i}=\frac{{\varphi }_{i}\cdot {k}_{i}}{\mathop{\sum }\limits_{j=1}^{n}{\varphi }_{j}\cdot {k}_{j}},$$

The model of^12^ is a *hybrid PA-fitness* growth model, in the extent that growth is both driven by the time-dynamic effect of degree (which varies at every step of the PA process, namely *k*_*i*_ = *k*_*i*_(*t*)) and by the static effect of fitness values, each assigned at the time a node is joining the network^13^. When node fitness is exclusively driven by node degree (*φ*_*i*_ = 1), the model is converted to the classic BA model shown in relation (1). Going beyond this consideration, the authors of^17^ argued that the underlying fitness distributions (instead of by default the degree) are directly responsible for the emergence of scale-free networks. Within this free-of-degree context, they defined the connecting probability between a new (*j*) and an existing (*i*) node proportionally to the intrinsic (non-negative) fitness *φ*_*i*_^13,17^, according to the relation:

3 $${p}_{i}={\varphi }_{i}/\mathop{\sum }\limits_{j=1}^{n}{\varphi }_{j}.$$

Therefore, in the model of^17^, node connections are developed with a probability that is proportional to the fitness of the participating nodes. This approach generates networks with PL degree distributions when the underlying node fitness distributions are also PL^13^. In general, the fitness model of^17^ proposes an alternative to the BA algorithm for generating scale-free topologies^18^, where node fitness represents the ability of nodes to compete for new connections. In real-world networks, fitness values are related to intrinsic qualities of the nodes (e.g. rank, wealth, population, size, etc.) and they generally represent the idea of competitive advantage within a competitive environment^18^. More lately, the authors of^19^ advanced the conceptualization of the fitness model by introducing the *Lognormal Fitness Attachment* (LNFA). In this model, the fitness values *φ*_*i*_ are multivariable functions instead of single (weight) values and they are defined as the product of a node’s intrinsic attributes^13,19^, according to the relation:

4 $${\varphi }_{i}=\prod _{p}{\varphi }_{ip},$$

where *p* is the number of attributes, whereas the connection probability is given by relation (3). When *p* is sufficiently large and includes statistically independent attributes, it is shown that node fitness is lognormally distributed regardless of the distribution type of the independent attributes^13,19^. The LNFA can better explain growth processes in real-world networks, where it is also likely for the latecomers to become hubs relatively quickly, due to the including tunable parameter (expressed by the shape of the lognormal distribution), which can generate various networks corresponding to different real-world contexts^13^.

Fitness-based models appear attractive in the relevant literature^13^, obviously due to their ability to generate SF networks based on either known fitness distributions or real-world fitness values. For instance, the authors of^20^ used a fitness model to test the generative mechanism of the World Trade Web, which describes the network defined by the trade relationships between countries worldwide. The analysis showed that the PL-distributed *Gross Domestic Product* (GDP) was in line with the topological fitness controlling the network growth and revealed an excellent agreement of topological features between the empirical and the fitness-based model. The authors of^21^ studied how different choices of fitness distributions and linking functions affect the SF property. In all cases, they found that the generation of SF networks is straightforward and thus that the SF property is indifferent to the initial choices. In an attempt to study the interplay between fitness and PA, the authors of^18^ examined a pair of hybrid fitness models, the first (model-A) growing with randomly added connections and the second one (model-B) under a degree-driven PA. The analysis showed that the degree distribution of model-A decays exponentially, whereas model-B shapes a PL pattern. In the work of^22^, the authors studied the effect of competition in the network growth when the fitness distribution is a PL. By including an exponent controlling the influence of fitness, they showed that the generated networks vary between the BA and the hybrid PA-fitness model of^12^. The authors of^19^ examined how variations of the parameters of the lognormal distribution affect the SF property in the generated network and they showed that such variations can recover both exponential and PL degree distributions. The PL exponents were found within the typical range describing real-world networks and they proposed their network-construct as a basis for new protocols enabling P2P networks to establish topologies contributing to search optimization.

Within the context of the evolving relevant research, the authors of^23^ surveyed the existing fitness-based models for generating SF networks, observing three major categories represented by the hybrid-fitness model of Bianconi and Barabsi^12^, the fitness model of Caldarelli *et al*.^17^, and the lognormal fitness model of Ghadge *et al*.^19^, as previously mentioned. In the work of^24^, the authors expanded the PA mechanism by assuming that the preference of nodes to connect is influenced by their indirect neighbors as well. They showed that their procedure (named *cyclic preferential attachment* - CPA) is broader than the traditional PA and more flexible in modeling real-world networks. The authors of^25^ developed a framework for the analysis of PA models based on the performance of the model parameters to control the degree distribution and the clustering coefficient. They also introduced a relevant parameterized model and showed that both PL degree distribution and clustering coefficient parameters are controllable. This work is among the first using the term *Generalized PA*, another reference of which can be found in the work of^26^, which develops a hybrid PA-fitness model to study the scientific citation process. In this model, the fitness value is the aging of scientific papers, which was chosen based on empirical observations. The analysis produced models satisfactorily explaining real-world citation networks. The authors of^27^ proposed a fitness-based model building on a game-theoretic attachment mechanism instead of on PA. The analysis showed that optimization to converge towards Nash equilibrium leads to the emergence of scale-free and small-world features. This approach appeared to better model networks ruled by high rationality. More recently, the authors of^13^ introduced an unconventional but also insightful fitness conceptualization. Their behavioral approach was ruled by the intuition that many actors participating in evolving networks do not have either high expectations or the means to connect with the most skillful actors in the network. For this community, the prime survival strategy is restricted to the more realistic goal of trying not to lose instead of getting the maximum utility. Within this context, the authors proposed a plausible fitness-based behavioral mechanism building on the *minimization of maximum exposure to node unfitness* (defined as the inverse of node fitness), which appears better in describing heterogeneous real-world supply-chains networks.

As is evident by the previous review, fitness-based models emerged in the literature in an attempt to produce SF models that better fit to empirical observations, according to which network growth seems to be driven by more forces than just by connectivity (node degree). Initially, fitness was introduced in a hybrid form, as a factor controlling the degree-driven PA process, while, at next, it was disengaged from PA and contributed to a broader conceptualization driving network growth^13,23^. However, the authors of^13^ observe that, although fitness-based models have shown that preferential attachment is not necessary to produce SF networks, the hybrid (i.e. PA-fitness) models remain attractive in the literature. This is because hybrid models can produce SF networks even when the underlying fitness distribution is not PL, which does not happen in the disengaged (i.e. fitness without degree-driven PA) models that result in SF networks only when the underlying fitness distribution is PL^13^. Moreover, in disengaged models, fitness seems to be more a static (or initial) configuration of node attractiveness rather than a time-dynamic process rearranging the fitness values at every step of network growth, as in the case of the degree-driven PA where node degrees are computed at every step of network growth. In particular, the authors of^17^ note that their *…model, as defined, is static, but it can straightforwardly be considered a dynamic one by adding new vertices at every time step and linking them to the existing ones according to the above attaching rule …*. Also, in the work of^22^, we can find an analysis of dynamic properties in a fitness-based network growth process, but this dynamic behavior is related to changes in the degree in time and not of the fitness values. Finally, as the authors of^13^ note, the …*concept of node fitness can be thought of as the amalgamation of all the attributes of a given node that contribute to its propensity to attract links. Indeed, one of these attributes could be the node degree, which would be a dynamic attribute that changes value as the network grows, whereas many other attributes of fitness would be static…*.

As it can be observed, the time dynamics of the non-degree-driven PA fitness models have not yet been studied in a comprehensive framework because current literature seems to conceptualize the time-dynamics of fitness in the context of adding new nodes in the growing network, where new fitness values have to be assigned to the newcomers to join the network. A bright attempt to overcome this restriction was made by the authors of^28^, who introduced a betweenness-driven instead of degree-driven PA growth process. In an attempt to better explain dynamics in social networks, the authors observed that degree is not the major attractor of new social links and thus the degree-driven PA cannot fully explain social network dynamics. Within this context, they introduced a PA model driven by weighted betweenness (the WBPA model), where the criterion for a node to connect is the weighted betweenness centrality instead of the degree. The WBPA model went beyond current fitness-based considerations to the extent that betweenness centrality was being computed at every step of network growth and therefore growth was defined by a time-dynamic topological fitness (i.e. weighted betweenness). This approach led to models more accurately describing a wide range of real-world social networks. The authors observed that node-betweenness is a better indicator of social attractiveness^28^ because it impels …*individuals to (intuitively) perceive node’s betweenness as the capacity of bridging communities, irrespective of its degree*….

Within this context, this paper goes beyond the work of^28^ and studies network growth under different aspects of time-dynamic topological fitness, namely where the fitness changes values, as the network grows. In particular, the study considers five different topological node-attributes as time-dynamic fitness of network growth: node-degree (*k*), clustering coefficient (*C*), betweenness centrality (*CB*), closeness centrality (*CC*), and eigenvector centrality (*CE*), which will be called control-attributes henceforth. The purpose of the research is twofold; first, to examine whether the networks grown under these different control-attributes are ruled by the SF property, and secondly, to detect topological differences and to compare the emerging topologies amongst these different types of generated networks. The further purpose of this paper is to contribute to the literature demand about studying time dynamics of fitness models, in a context that is free of the degree-driven PA and broader than the dynamics caused by adding new nodes in the growing network. The remainder of this paper is organized as follows; Section 2 describes the methods and the models’ construction, Section 3 detects the SF property and compares major topological attributes in the resulting networks, and, finally, Section 4 addresses the conclusions.

## Methods

### Model construction

The model construction algorithm builds on the uniform attachment algorithm of the *BA* model^2^, which is customized accordingly, so that network growth to be driven by one control attribute (*X*). Details of the algorithm and the coding are available in the Appendix. Graphs (null-models) generated by this procedure are undirected and unweighted. In total, 150 undirected null-models are constructed, divided into 5 families (groups) {*G*(*C*), *G*(*CB*), *G*(*CC*), *G*(*CE*)}, each corresponding to a control attribute. Within each family *G*(*X*), where *X* = *k*, *C*, *CB*, *CC*, and *CE*, 30 null-models are included. This number was chosen to apply statistical inference techniques referring to the normal distribution^29^. In particular, the *G*(*k*) family includes fitness models grown under the control of *degree* (*k*), *G*(*C*) under the control of *clustering coefficient* (*C*), *G*(*CB*) under the control of *betweenness centrality* (*CB*), *G*(*CC*) under the control of *closeness centrality* (*CC*), and *G*(*CE*) under the control *eigenvector centrality* (*CE*), respectively. The number of nodes in each family is typical and ranges from *n* = 50 to *n* = 1500 with a lag of 50 nodes (*n* = 50, 100, 150, 200, …, 1450, 1500). Null-models participating in the analysis are shown in the Appendix. Null-models with more 1500 nodes were not generated due to the time-complexity of the betweenness-driven and closeness-driven model construction. Reducing the algorithm’s complexity suggests an avenue for further research.

### Topological analysis

The topological network analysis consists of three parts. The first examines the degree distributions *p*(*k*) of the available null-models. The overall examination is done graphically, descriptively, and through statistical interference. In the graphical approach, the degree distributions of the available null-models are plotted to 3-dimension (3d) bar-charts^30^. In these bar-charts, the *x*-axis represents the node degrees, the *y*-axis represents the ranking of the null-models arranged into ascending order, and the *z*-axis represents the frequencies *n*(*k*) of nodes having degree *k*. Corresponding axes along model families have a fixed scale to facilitate comparisons. Next, the descriptive approach builds on the construction of boxplots, which are box and whisker plots enclosing the interquartile range of the data in a box, which has the median displayed within^29^. Boxplots are used to display information about location, variability, and asymmetry of the degree distributions configured by the available 30 null-models for each degree-class and a certain null-model family. Finally, the statistical inference approach builds on parametric fitting and the construction of 95% confidence intervals (CIs). At first, for each of the available 30 null-models (included within a certain family) a PL curve^29^ is fitted to the degree distribution data. Isolated nodes are not taken under consideration in these PL-fittings. Next, 95% CIs are constructed on the available sets of (30 in number) PL-exponents and (also 30 in number) coefficients of determination (*R*^2^) resulted from the previous fittings. The CIs are then compared between the null-model families to detect statistical differences.

The second part of the analysis examines differences in topological layouts of equal-size null-models throughout the available families. Network topologies are embedded in the 2d-Euclidean space and are visualized using the Force-Atlas layout, which is available in the open-source software of^31^. This layout is generated by a force-directed algorithm (see^32^), which is used in its default parameters. This algorithm applies repulsion strengths between network hubs while it arranges hubs’ connections into surrounding clusters. Graph models represented in this layout have therefore their hubs centered and mutually distant (i.e. the distance between hubs is as highest as possible) in the topological map, whereas nodes of lower degree are placed as closely as possible to their hubs^8^.

The third part of the analysis examines differences of topological measures, metrics, and statistics amongst the available null-model families. Provided that each measure captures a certain aspect of network topology^4,8^, a variety of measures are examined to better approximate network topology of each family in total. The network measures participating in this analysis are shown in Table 1.

**Table 1:** Network measures participating in the topological analysis.

Measure	Description	Mathematical Expression	Reference(s)
*Network*	A graph expressed as the pair set of nodes *V* and edges *E*.	*G*(*V*,*E*)	^33^
*Network edges* (*m*)	The number of links included in the network	*m* = |*E* | = card(*E*)	^33^
*Diagonal Distance* (*dd*)	The average distance of the non-zero elements from the main diagonal of the network’s adjacency.	$dd(G)=\frac{1}{\sqrt{2}\cdot {n}^{2}}\sum _{(i,j)\in E}|i-j|$	^8^
*Network diameter d*(*G*)	The longest path in the network.	$dG=\,{\rm{\max }}\{d(i,j)|i,j\in V\}$	^33^
*Node Degree* (*k*)	The number of edges being adjacent to a node.	$$\begin{array}{c}{k}_{i}=k(i)=\sum _{j\in V}{\delta }_{ij},\,{\rm{where}}\\ {\delta }_{ij}=\{\begin{array}{c}1,\,{\rm{if}}\,{e}_{ij}\in E\\ 0,\,{\rm{otherwise}}\end{array}\end{array}$$	^1,33^
*Maximum degree* (*k*_max_)	The maximum degree of the network nodes.	${k}_{\max }=\,\max \,\{k(i)\in V|i=1,2,\mathrm{..}.,n\}$	^33^
*Isolated nodes* (*k*_o_)	The number of unconnected (*k* = 0) nodes in the network.	${k}_{o}=card\{k(i)=0|i=1,2,\mathrm{..}.,n\}$	^33^
*UDV*	*Unique degree values*: the number of distinct degrees considered for computing the degree distribution of a network.	n/a	In this paper
*Hubs*	The number of network nodes with a degree within the last fifth of the degree-range.	$Hubs=\{i\in V|k(i)\ge {k}_{\max }-(\frac{{k}_{\max }-{k}_{\min }}{5})\}$	In this paper
*Average Path Length* $\langle l\rangle$	Average network shortest path lengths *d*(*i*,*j*).	$\langle l\rangle =\frac{\sum _{v\in V}d({v}_{i},{v}_{j})}{n\cdot (n-1)}$	^11,33^
*COM*	The number of connected components in the network.		^8,34^
*Assortativity* (*r*)	A measure of nodes’ preference to attach to other similar nodes, where *e*_*jk*_ is the joint probability distribution of the remaining degrees of two nodes at either end of a randomly chosen end.	$$\begin{array}{c}r=\frac{1}{{\sigma }_{q}^{2}}\sum _{jk}jk({e}_{jk}-{q}_{j}{q}_{k}),\\ {\rm{where}}\,\sum _{jk}{e}_{jk}=1\,{\rm{and}}\,\sum _{j}{e}_{jk}={q}_{k}\end{array}$$	^8,35^
*Local Clustering Coefficient* (*C*(*i*))	The number of a node’s connected neighbors *E*(*i*), divided by the number of the total triplets *k*_*i*_(*k*_*i*_–1) shaped by the node.	$C(i)=\frac{E(i)}{{k}_{i}\cdot ({k}_{i}-1)}$	^11^
*Modularity* (*Q*)	Objective function measuring the potential of a network to be subdivided into communities, where *g*_*i*_ is the community of node *i*, [*A*_*ij*_ – *P*_*ij*_] is the actual minus the expected number of edges falling between a particular pair of nodes.	$$\begin{array}{c}Q=\frac{\sum _{i,j}[{A}_{ij}-{P}_{ij}]\cdot \delta ({g}_{i},{g}_{j})}{2m},\\ {\rm{where}}\,{\delta }_{ij}=\{\begin{array}{c}1,\,{\rm{if}}\,{g}_{i}={g}_{j}\\ 0,\,{\rm{otherwise}}\end{array}\end{array}$$	^6^
*ω-index*	Index detecting whether a network has the small-world property, or lattice-like, or random-like characteristics.	$\omega =\frac{{\langle l\rangle }_{rand}}{\langle l\rangle }-\frac{\langle C\rangle }{{\langle C\rangle }_{latt}}$	^36^
*COI*	*City organization index*: small values (≈0) express that the network is described by a well-organized pattern. Values close to one (≈1) express deficiency in organization and planning.	${r}_{n}=\frac{n(1)+n(3)}{{\sum }_{k\ne 2}n(k)}$	^37^

Each measure shown in Table 1 is computed for all available 30 null-models within a certain family. Next, 95% CIs^29^ are constructed on the available sets of measures (each set includes 30 values of a single network measure) and then comparisons are made amongst family CIs, for each measure. Such comparisons can indicate statistically maximum and minimum performances for each family and per measure. Therefore, based on relevant literature^1,6,8,11,33–37^, we can evaluate which extremum (min or max) behavior is desirable in a network structure and thus which extremum suggests an optimum performance for a certain network attribute. For instance, an increasing average degree is desirable to the extent that it improves network connectivity^3,4,33^ and thus the maximum is the optimum performance for this measure. In contrast, constructing networks of long diameter is not desirable for directness^3,33^ and thus the optimization goal for this measure is to reach the minimum. This conceptualization allows constructing a comparative directed graph, where each family is assigned to a node and thus a directed connection *i* → *j* ≡ (*i*,*j*)≡*e*_*ij*_ may express that family *X* = *i* outperforms family *X* = *j* in terms of measure *y*, according to the relation:

5 $$i\to j\equiv {e}_{ij}\in E|{\rm{CI}}(i|y)\mathop{ > }\limits^{{\rm{outperforms}}}{\rm{CI}}(j|y),$$

where CI(*i* | y) is a 95% CI of family *X* = *i* for the measure *y*. Therefore, the weighted out-degree of this comparative directed graph will indicate the family that is an out-degree hub, namely the family that has a desirable performance in more topological measures (see Table 1) than the other families. This approach may detect the topology that can be loosely considered as better, namely to the extent that it outperforms in more topological measures the other families.

## Results

### Examination of the SF property

**Figure 1:**
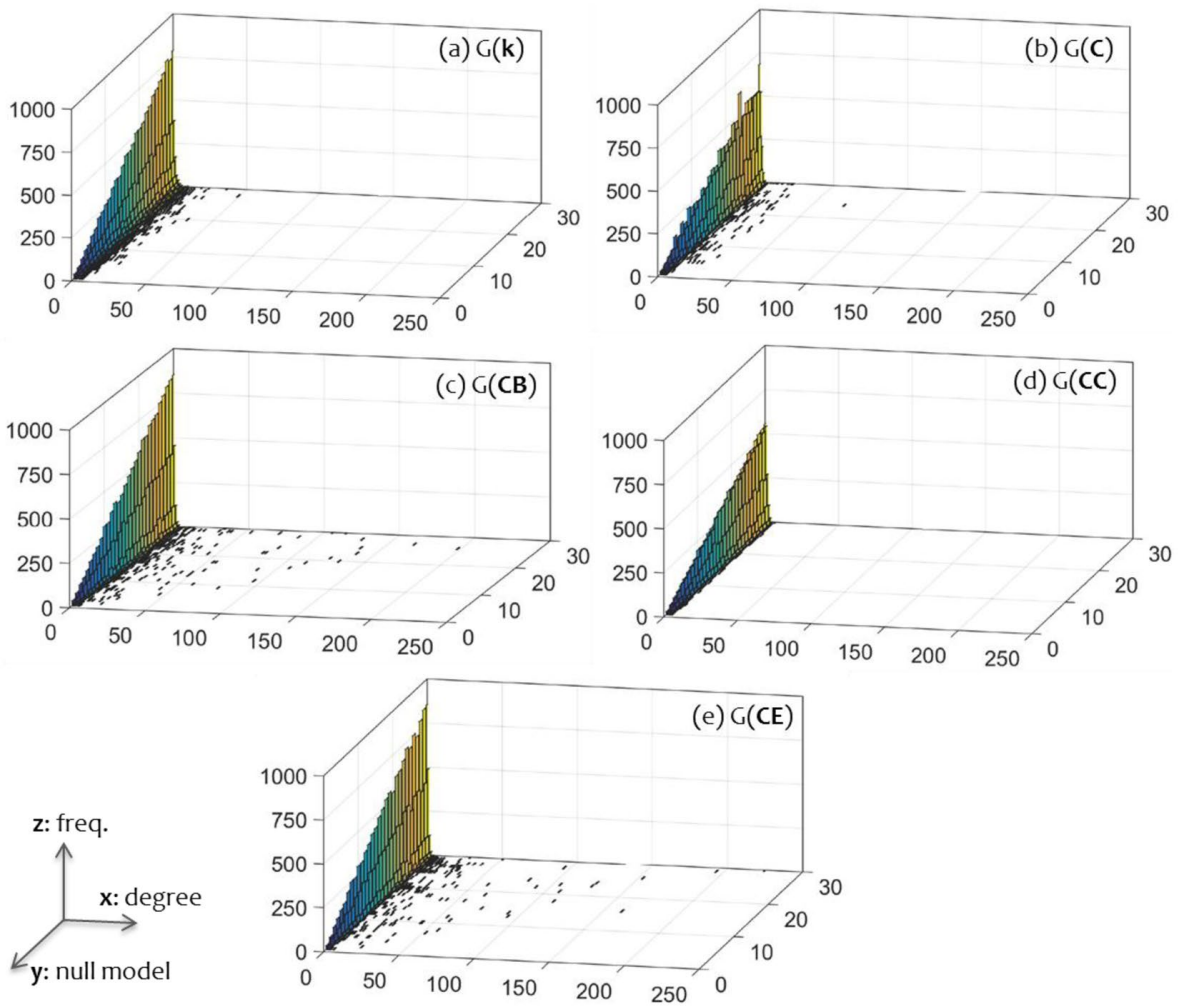
Three-dimension (3d) bar-charts illustrating the degree distributions of the null-model families generated under the control (**a**) of degree *G*(*k*), (**b**) clustering coefficient *G*(*C*), (**c**) betweenness centrality *G*(*CB*), (**d**) closeness centrality *G*(*CC*), and (**e**) eigenvector centrality *G*(*CE*). The *x*-axis represents node degree, the *y*-axis the ranking of the null-models arranged into ascending order, and the *z*-axis the frequencies *n*(*k*) of nodes having degree *k*.

Degree distributions are plotted to the 3d bar-charts of Fig. 1. In total, the available 150 in number degree distributions range from 4 up to 21 cases (including *k* = 0), which in particular range between 6–17 cases for *G*(*k*) family, between 5–15 cases for *G*(*C*), between 5–19 cases for *G*(*CB*), between 5–7 cases for *G*(*CC*), and between 4–21 cases for *G*(*CE*), respectively. As it can be observed, frequencies in each family are descending and shaping PL-like patterns. Among the available families, betweenness *G*(*CB*) and eigenvector centrality *G*(*CE*) have the most long-tailed distributions, whereas closeness centrality *G*(*CB*) has the shortest-tailed degree distributions.

**Figure 2:**
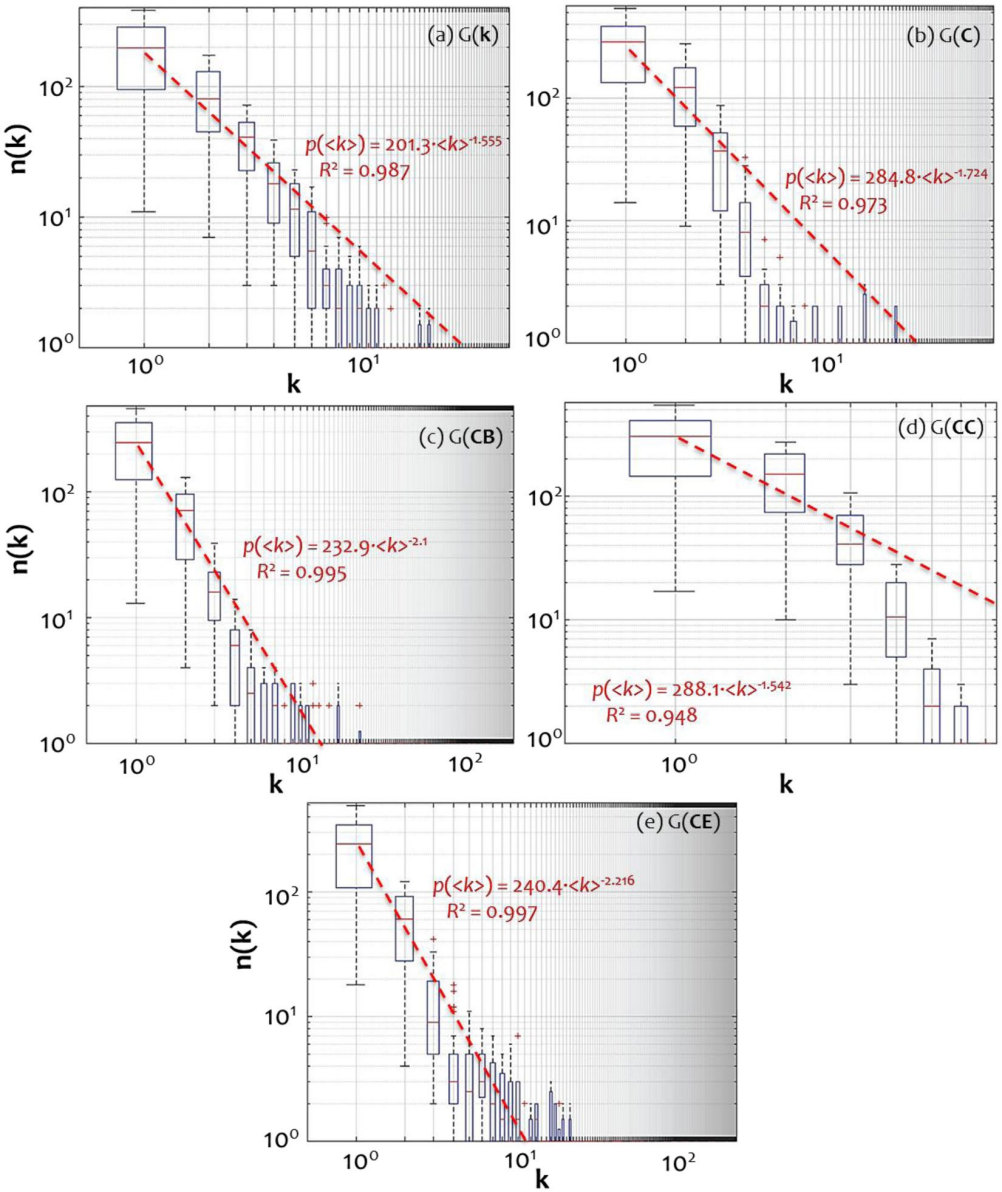
Boxplots showing how degree distributions of the available 30 models that are included in the families of (**a**) of degree *G*(*k*), (**b**) clustering coefficient *G*(*C*), (**c**) betweenness centrality *G*(*CB*), (**d**) closeness centrality *G*(*CC*), and (**e**) eigenvector centrality *G*(*CE*) are distributed along the degrees. The PL curves are fitted to average values per node degree (log-log axes are used).

A similar to Fig. 1 picture can be shaped by the examination of the boxplots shown in Fig. 2. In this figure, boxplots show how degree distributions of the available 30 models that are included in each of the null-model families {*G*(*k*), *G*(*C*), *G*(*CB*), *G*(*CC*), *G*(*CE*)} are distributed along the degrees. For instance, the first boxplot in Fig. 2a shows how the frequencies of nodes having degree *k* = 10°=1 are distributed throughout the 30 members of the *G*(*k*) family. In these log-log representations, the boxplots’ arrangement shows a linear descending trend providing indications that degree distributions in each family follow a PL pattern. This observation is verified by the PL fittings applied to averages per degree, where all cases are described by very high determination (*R*^2^_*X*_ ≥ 0.948). However, only the PL exponents of the betweenness (Fig. 2b) and eigenvector centrality (Fig. 2b) families range within the typical (empirical) interval 2<*γ* < 3^1^, implying a better performance of these families in terms of scale-freeness.

**Figure 3:**
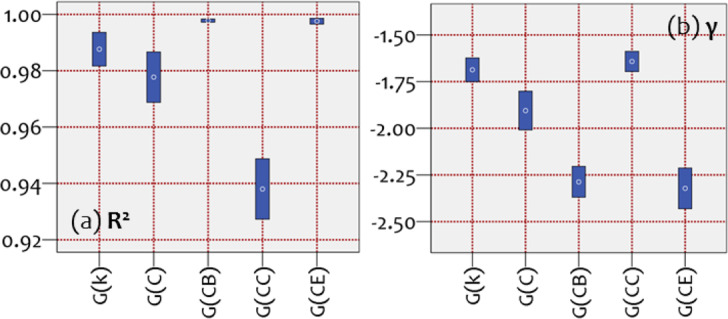
95% confidence intervals (CIs) of the average (**a**) coefficient of determination (R^2^) and (**b**) power-law (PL) exponent (γ) of the PL-fittings, computed within each family of networks G(k), G(C), G(CB), G(CC), and G(CE). Measures k, C, CB, CC, and CE within parentheses express the attribute controlling time-dynamic topological fitness.

Further, a statistical inference analysis is applied to examine the SF property of each family, as shown in Fig. 3. The 95% CIs are constructed on sets of the gamma PL exponents (*γ*) and coefficients of determination (*R*^2^) resulted from the PL fittings. Despite the insufficient data describing cases of small networks (*n* ≤ 100), all *R*^2^ CIs in Fig. 3a appear very high. In particular, CIs of *R*^2^ are ${{\rm{CI}}}_{{R}^{2}}(k)$=[0.937, 0.998], ${{\rm{CI}}}_{{R}^{2}}(C)$=[0.892, 0.997], ${{\rm{CI}}}_{{R}^{2}}(CB)$=[0.994, 0.999], ${{\rm{CI}}}_{{R}^{2}}(CC)$=[0.874, 0.996], and ${{\rm{CI}}}_{{R}^{2}}(CE)$=[0.987, 0.999], respectively. The respective average values are *R*_*k*_^2^ = 0.988, *R*_*C*_^2^ = 0.977, *R*_*CB*_^2^ = 0.998, *R*_*CC*_^2^ = 0.937, and *R*_*CE*_^2^ = 0.996 (Fig. 3a). As it can be observed, the average coefficients of determination (<*R*^2^ > ) of the PL-fittings are sufficiently high (>0.92) for all the available families. Overall, the *R*^2^ CIs in Fig. 3a illustrate that all null-model families have their degree distributions satisfactorily described by PL patterns and thus all families are ruled by the SF property. Further, Fig. 3b shows that the average *γ* (gamma) PL exponents are close to the typical interval 2<*γ* < 3 describing real-world SF networks^1^. Moreover, the CIs of betweenness *G*(*CB*) and eigenvector centrality *G*(*CE*) families are included in this typical interval. This implies a better performance of these cases, in terms of scale-freeness, verifying the previous observations.

Overall, the degree distribution analysis shows that all available null-models grown with time-dynamic topological fitness (i.e. where fitness is a dynamic attribute that changes value as the network grows) under the control of degree (*k*), clustering coefficient (*C*), betweenness (*CB*), closeness (*CC*), and eigenvector centrality (*CE*) have the SF property. In the case where fitness is controlled by degree, this result is expected and complies with the literature^13,17,23^, because the generated models are equivalent to BA models. Also, in the case of betweenness, the results are in line with the findings of^28^. However, for the remaining cases (*C*, *CC*, *CE*), the results advance the existing literature (which states that fitness-based models generate SF networks only when the underlying fitness distribution is a PL) by revealing that time-dynamic topological fitness can generate SF networks even when the underlying fitness distribution is not SF (it is uniform, see Appendix).

### Topological analysis

**Figure 4:**
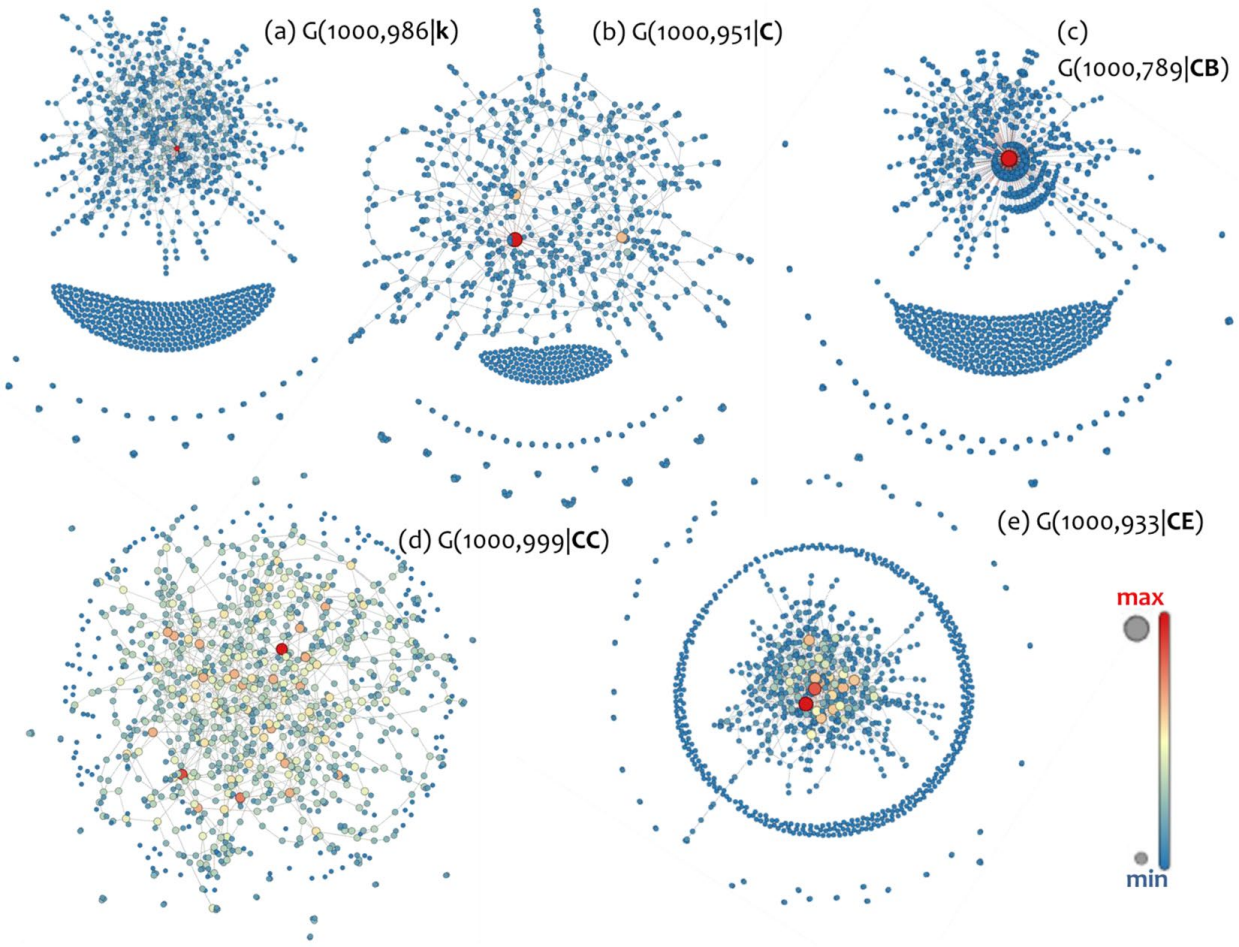
Topological layouts of equal-size (*n* = 1000) null-models *G*(*n*,*m* | *X*), where *n* is the number of nodes, *m* the number of links, and *X* is the time-dynamic topological fitness controlling network growth. The null-model families shown in each case are (**a**) degree (*X* = *k*, *m* = 986), (**b**) clustering coefficient (*X* = *C*, *m* = 951), (**c**) betweenness centrality (*X* = *CB*, *m* = 789), (**d**) closeness centrality (*X* = *CC*, *m* = 999), and (**e**) eigenvector centrality (*X* = *CE*, *m* = 933). Layouts are visualized by using the Force-Atlas embedding available in the open-source software of^31^. Node color (from blue to red) and size (from small to big) are proportional to node degree.

This part examines differences in topological layouts of equal-size null-models amongst the available families. A representative picture of the topologies produced by the proposed growth model of time-dynamic topological fitness is shown in Fig. 4, where null-models of size *n* = 1000nodes are displayed. As it can be observed, the topological layouts appear quite different amongst these five families. In particular, the betweenness centrality *G*(*CB*) layout configures a mono-centric pattern that is similar to a superstar network described by the authors of^33^. This layout is described by a dominant hub, a nodes’ concentration radially to the hub, and a cluster of isolated nodes eccentrically arranged. On the contrary, the closeness centrality *G*(*CC*) layout shapes a polycentric pattern, where hubs are mutually distant and all the other nodes are scattered throughout the network space into a mesh-like arrangement. The degree *G*(*k*) and eigenvector centrality *G*(*CE*) layouts configure core concentrations, but they considerably differ from the superstar pattern of *G*(*CB*). However, the *G*(*CE*) layout is more polycentric then *G*(*k*) and has hubs with a higher degree (denoted by node size). Also, the arrangements of isolated nodes in these two layouts are different. Overall, layouts shown in Fig. 4 vary from a mesh-like of *G*(*CC*) to a superstar-like pattern of *G*(*CB*), following the ordering *G*(*CC*), *G*(*C*), *G*(*CE*), *G*(*k*), and *G*(*CB*), respectively.

**Figure 5:**
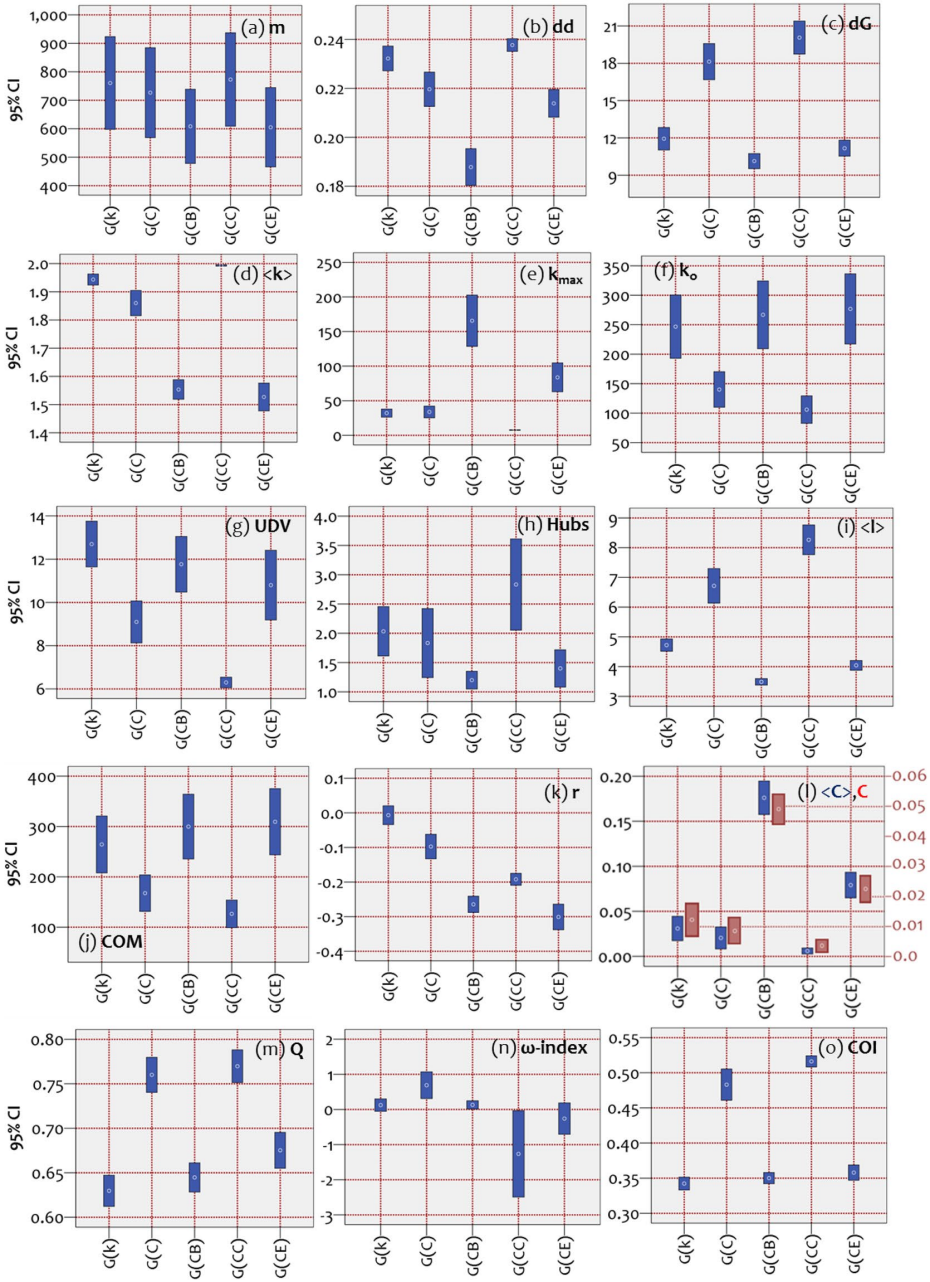
95% confidence intervals (CIs) of the average (**a**) *number of links* (*m*), (**b**) *diagonal distance* (dd, see^8^), (**c**) *network diameter* (dG), (**d**) *average degree* (<k > ), (**e**) max degree (k_max_), (**f**) number of isolated nodes (k_o_), (**g**) unique degree values (UDV), (**h**) number of hubs (Hubs), (**i**) average path length (<l > ), (**j**) number of connected components (COM), (**k**) network assortativity (**r**), (**l**) average and global clustering coefficient (<C > , C), (**m**) modularity, (**n**) ω-index (ω), which was proposed by^36^, and (**o**) city organization index (COI), which was proposed by^37^. All CIs were computed for the members of each network family G(X) = {G(k), G(C), G(CB), G(CC), G(CE)}. Measures X = k, C, CB, CC, and CE express the attribute controlling growth in the time-dynamic topological fitness algorithm.

Next, the results of the analysis based on statistical inference of network topology measures are shown in Fig. 5. Each CI (95%) corresponds to a certain family and a certain measure. Non-overlaid intervals between compared cases indicate statistical differences, which imply that displayed differences are only 5% likely to be a matter of chance. As it can be observed, in the majority of cases, the CIs shown in Fig. 5 do not overlay. This generally implies that the network topologies amongst the available model families differ in many aspects. An exception to this observation is the number of links (Fig. 5a), where all cases can be considered statistically equal. In all other cases, 95% CIs of the examined null-model families statistically differ. In particular, Fig. 5b shows that the diagonal distances (dd)^8^ of the model families are statistically different. The dd is a spectral metric, which was proposed by the author of^8^ and measures the average distance from the main diagonal of non-zero elements of the network’s adjacency matrix. Differences in dd reveal topological differences between networks that sometimes cannot be observed in the degree distributions of the networks, such as in cases when the degree distributions are the same^8^. Within this context, statistical differences captured in Fig. 5b reveal topological differences in the sparsity patterns of the network adjacencies amongst the available families.

In particular, the CI of *G*(*CC*) for the measure of dd implies that models of this family are mostly scattered in their adjacency matrices, whereas *G*(*CB*) implies that models of this family have the most concentrated arrangement along the main diagonal of their adjacency. These observations comply with the polycentric and star-like patterns previously observed in the topological layouts of these families, respectively. Next, Fig. 5c shows that betweenness centrality family has (on average) the shortest network diameter (although not statistically different than *G*(*CE*)), whereas *G*(*CC*) has the largest one (although not statistically different than *G*(*C*)). Further, Fig. 5d shows that *G*(*CC*) has the highest average degree, whereas families of betweenness *G*(*CB*) and eigenvector centrality *G*(*CE*) have the smallest. This complies with the observation in the topological layouts (Fig. 4), according to which the topologies of *G*(*CB*) and *G*(*CE*) are more hub-and-spoke-like, in contrast with the more mesh-like topology of *G*(*CC*). A degree-based consideration is also made in Figs. 5e and 5f. In particular, Fig. 5e shows that betweenness centrality family *G*(*CB*) has the maximum degree, which complies with the superstar-like observation made in Fig. 4. The Fig. 5f shows that the clustering coefficient *G*(*C*) and closeness centrality *G*(*CC*) families have the shortest number of isolated nodes, which complies with their mesh-like topologies observed in Fig. 4. Next, Fig. 5g shows that closeness centrality family *G*(*CC*) has less unique degree-values than the others, implying that it has the least long-tailed degree distribution (and thus relatively more hubs than the other families). This complies with the mesh-like topology of *G*(*CC*) observed in Fig. 4. Also, Fig. 5h illustrates the superstar-like topology of the betweenness centrality family *G*(*CB*) since its CI [1.05,1.35] is considerably close to one (implying the existence of one hub in the majority of models within this family). Next, Fig. 5i shows that the closeness centrality *G*(*CC*) family has the longest average path length, which complies with the mesh-like topology observed in Fig. 4. Next, Fig. 5j shows the number of components in the network, which is similar to the case of isolated nodes in Fig. 5f. Further, Fig. 5k shows that the degree (BA models) family *G*(*k*) has the highest assortativity, implying a good tendency of nodes to attach with similar ones. Next, Fig. 5l shows that the betweenness centrality family *G*(*CB*) has the highest clustering coefficient. An interesting observation is that growth under the time-dynamic topological fitness of the clustering coefficient (*X* = *C*) generates networks of low clustering (*G*(*CB*)) but not the lowest (the eigenvector centrality family has the lowest one).

Next, Fig. 5m shows that families of clustering coefficient *G*(*C*) and closeness centrality *G*(*CC*) have the best tendency to be divided into communities. This result complies with their mesh-like topologies observed in Fig. 4. In Fig. 5n, CIs closer to the zero-line illustrate small-world-like (SW-like) characteristics, where positive scores imply randomness and negative scores lattice-like characteristics^38^. Within this context, families of betweenness centrality *G*(*CB*) and degree *G*(*k*) have SW-like characteristics with random trends, family *G*(*C*) has random-like characteristics, family *G*(*E*) has lattice-like characteristics with SW-like trends, and *G*(*CC*) has lattice-like characteristics. These results comply with the mesh-like topology of *G*(*CC*), the superstar-like topology of *G*(*CB*), and the intermediate topological layouts observed in Fig. 4. Finally, Fig. 5o shows which family has a better-organized structure, in the context of considering a network as a city and evaluating its topology in terms of the number of incomplete crossovers and dead ends^37^. According to this metric, the families of degree *G*(*k*), betweenness centrality *G*(*CB*), and eigenvector centrality *G*(*CE*) have the most well-organized patterns.

Overall, the statistical inference analysis illustrates that many topological aspects differ amongst the available null-model families. This implies that networks generated under time-dynamic topological fitness considerably differ in their topological attributes. To summarize all findings, we construct Table 2, showing cases of minimum or maximum performance, which are extracted from Fig. 3 and Fig. 5 and are compared with the desired network performance noted as optimum.

**Table 2:** Summary of measures with minimum or maximum CIs^(a)^.

Measure	Optimum condition^(b)^	Null-model family				
		*G*(*k*)	*G*(*C*)	*G*(*CB*)	*G*(*CC*)	*G*(*CE*)
Dd	n/a			min		
<*k* >	max			min	max^*^	min
*r*	min	max		min^*^		min^*^
*k*_max_	max			max^*^	min	
*k*_o_	min		min^*^		min^*^	
UDV	max				min	
*γ*	2 <*γ* < 3			min^*^		min^*^
*R*^2^	→ 1				min	
*ω*-index	→0	^*^	max	^*^	min	min
COI	→ 0	min^*^		min^*^	max	min^*^
d*G*	min			min^*^		min^*^
*Q*	min	min^*^		min^*^		min^*^
COM	min		min^*^		min^*^	
<*C* >	max			max^*^	min	
*C*	max		min	max^*^	min	
<*l* >	min			min^*^	max	
Min		2	2	8	8	7
Max		1	2	3	3	0
Optimums^*^		2	2	8	4	5

a. Based on the analysis shown in Fig. 2 and Fig. 5.

b. Defined by the physical meaning of each measure, based on relevant literature (see Table 1).

**Figure 6:**
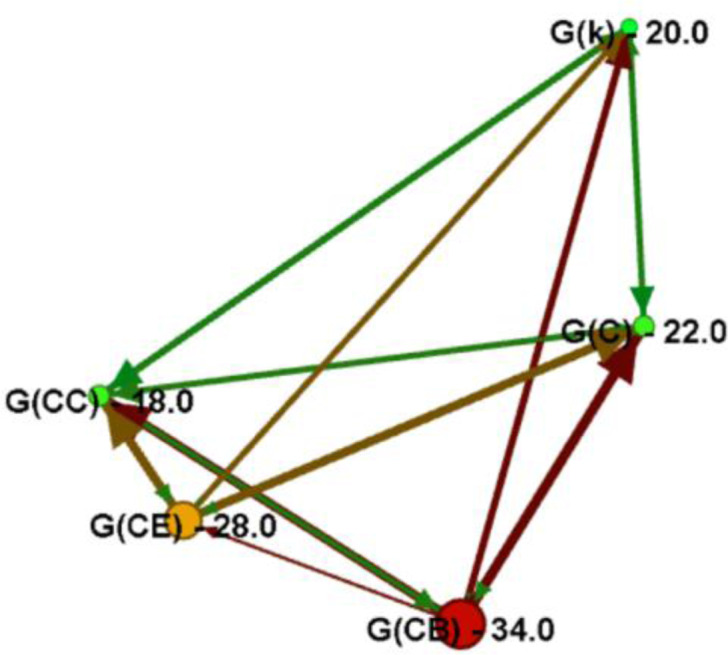
Comparative directed graph modeling the topological importance (weighted out-degree) of each family {*G*(*k*), *G*(*C*), *G*(*CB*), *G*(*CC*), *G*(*CE*)}, as expressed by different topological aspects shown in Table 2. The connectivity rule of the graph construction is shown in relation (5), where a directed link (*i*,*j*) of weight one (*w*_*ij*_ = 1) expresses that node *i* outperforms *j* in one measure shown in Table 2. Nodes are colored and sized proportionally to the weighted out-degree, where higher values indicate nodes outperforming in more topological attributes.

Further, based on data of Table 2, the comparative directed graph shown in Fig. 6 is constructed, according to relation (5). In this graph, the weighted out-degree indicates the topology that can be loosely considered as better, to the extent that it outperforms in more topological measures than the other families. As it can be observed in Fig. 6, the betweenness centrality family *G*(*CB*) is the weighted out-degree hub in the graph, being followed by the families *G*(*CE*), *G*(*C*), *G*(*k*), and *G*(*CC*), in descending order.

Overall, the previous analysis shows that null-models grown with time-dynamic topological fitness under the control of betweenness centrality (*X* = *CB*) outperform in more topological aspects the models belonging to the other families. This finding complies with the finding of the authors of ^28^, who observed that the node-betweenness suggests a better indicator of social attractiveness and with the observation of the authors of ^35^, who noted that superstar SF networks (describing the betweenness centrality family) are of the better topology of the BA model (describing the family of degree).

## Conclusions

In current literature, the fitness model has not yet been studied in a comprehensive context because most models are static and are restricting the model’s ability in generating scale-free (SF) networks only when the underlying fitness distribution is power-law. Aiming to broaden the time-dynamic conceptualization of fitness, this paper studied scale-free networks generated under time-dynamic topological fitness that changes as the network grows. Five different network attributes controlling topological fitness were taken into consideration; degree (leading to Barabasi-Albert models), clustering coefficient, betweenness, closeness, and eigenvector centrality. The analysis built on network analysis and statistical mechanics and examined the degree distributions of the generated null-models and compared topological aspects between families defined by each of the fitness attributes. The results showed, first, that growth under time-dynamic topological fitness appears indifferent to the underlying fitness distribution because all families included models with degree distributions very well fitted to power-law (PL) curves. Moreover, PL exponents of all families were close to the typical interval 2<*γ* < 3 describing real-world SF networks, while families of betweenness *G*(*CB*) and eigenvector centrality *G*(*CE*) were included within this interval. The examination of topological layouts showed that the topology of models generated under different topological fitness ranges from a mesh-like (describing the closeness centrality family) to a superstar-like (describing the betweenness centrality family) pattern. Moreover, all families appeared to have distinct topological layouts ranging from mesh-like to superstar-like patterns, following the ordering *G*(*CC*), *G*(*C*), *G*(*CE*), *G*(*k*), and *G*(*CB*), respectively. Finally, based on statistical inference of network topological aspects, the analysis showed that networks grown under the control of betweenness centrality outperform the others in scale-freeness and the majority of the other topological attributes. The overall results complied with the literature and with a recent work examining betweenness centrality as a social attractor. The overall approach attempted to broaden the conceptualization of fitness to a more time-dynamic context and provided evidence to disconnect the SF property from the underlying fitness distribution in the fitness-based models.

## Supplementary information

Supplementary files.

Supplementary data 1.

Supplementary data 1.

Supplementary data 1.

Supplementary data 1.

Supplementary data 1.

Supplementary data 1.

## Appendix

A1. Models’ construction algorithm

For a network *G*(*V*,*E*), expressed by the pair-set of nodes *V* and links *E*, we define *n* the number of nodes ($|V|=n$) and *m* the number of links ($|V|=m$).

The input parameters of the generative algorithm are:

*n*: the desired number of nodes in the network,

*m*_o_: the (constant) number of links added at every step of the growth,

*t*_*j*_: the *j*-th step of network growth,

*X*:the control attribute *X*={degree (*k*), clustering coefficient (*C*), betweenness centrality (*CB*), closeness centrality (*CC*), and eigenvector centrality (*CE*)} driving the attachment procedure.

All models are grown in discrete time (*t*∈${\mathbb{N}}$) that is set equal to the number of nodes (*t*_*n*_=*n*).

The algorithm is subjected to the following restrictions^31^:

$$\{\begin{array}{c}n > 0\\ 0 < {m}_{o} < n\\ 0 < m\le {m}_{o}\end{array}$$(A1.1).

The number of links added at every step of growth is chosen to one (*m*_o_=1).

Within this context, the construction algorithm of the time-dynamic fitness models is described as follows:

▪ At time zero (*t*_o_=0), *n* (in number) isolated nodes are generated in the network. At this time, a constant fitness value (*φ*_*i*_(*t*_o_)=*φ*_*i*_(0)=*φ*_o_) equal to 1/*n* (*φ*_o_=1/*n*) is assigned to all available isolated nodes. Therefore, at time zero, all nodes have the same (uniform) probability to connect.

▪ At the first step of growth (*t*_1_=1), *m*_o_ links are randomly added to the network. At this step, the topological measure corresponding to control-attribute *X* is computed for the current network.

The resulting scores *x*_*i*_(*t*_1_=1) of control-attribute *X* are then converted to current (*t*_1_=1) topological-fitness probabilities *x*_*i*_(1)^*^, according to the relation:

A1.2 $${x}_{i}{(1)}^{\ast }=\{\begin{array}{c}{x}_{i}(1)/\sum _{i}{x}_{i}(1),{x}_{i}{(1)}^{\ast }\in {\boldsymbol{{\mathbb{R}}}}\\ 0,{x}_{i}{(1)}^{\ast }\notin {\boldsymbol{{\mathbb{R}}}}\end{array}$$

where *i*=1,…,*n* is a network node.

At next, the new fitness values *φ*_*i*_(1) at growth-time *t*_1_ are computed by the sum of past fitness values *φ*_*i*_(0) and of current (*t*_1_=1) topological-fitness probabilities *x*_*i*_(1)^*^, according to the relation:

*φ*_*i*_(1) = *φ*_*i*_(0) + *x*_*i*_(1)^*^(A1.3).

▪ At the second step of growth (*t*_2_=2), another *m*_o_ links are added to the network proportionally-randomly to the ϕ (1) fitness-values and the procedure is repeated accordingly.

▪ At the *j*-th step of growth (*t*_*j*_=*j*), another *m*_o_ links are added to the network proportionally-randomly to the ϕ (j) fitness-values and the procedure is repeated accordingly. The resulting scores *x*_*i*_(*j*) of control-attribute *X* are converted to current (*t*_*j*_=*j*) topological-fitness probabilities *x*_*i*_(*j*)^*^, according to the relation:

A1.4 $${x}_{i}{(j)}^{\ast }=\{\begin{array}{c}{x}_{i}(j)/\sum _{i}{x}_{i}(j),{x}_{i}{(j)}^{\ast }\in {\boldsymbol{{\mathbb{R}}}}\\ 0,{x}_{i}{(j)}^{\ast }\notin {\boldsymbol{{\mathbb{R}}}}\end{array}$$

with *i*=1,…,*n*, and the new fitness values *φ*_*i*_(*j*) at growth-time *t*_*j*_=*j* are computed according to the relation:

*φ*_*i*_(*j*) = *φ*_*i*_(*j*–1) + *x*_*i*_(*j*)^*^(A1.5).

▪ The algorithm then terminates at the *n*-th step of growth (*t*_*n*_=*n*).

The models generated by this algorithm are undirected graphs.

Models *G*_*p*_(*X*), which are generated under the control of degree (*X*=*k*), are equivalent to *BA* models.

A2. Coding

The code of the models’ construction algorithm proposed in this paper is written in MATLAB (*m*-file)^30^ and is available as follows (other functions included in this code are available by the Brain Connectivity Toolbox at https://sites.google.com/site/bctnet/measures/list and provided as supplementary material)

function [ADJ] = tvt_fitness(n, mo, c)

% TIME VARIANT TOPOLOGICAL (TVT) FITNESS: This function generates a graph grown with time-variant topological fitness under the control of degree(k), clustering coefficient (c), betweenness centrality (cb), closeness centrality (cc), and eigenvector centrality (ce).

%

% INPUTS

% n: number of nodes at the final network,

% mo: number of links added with every new node,

% c: control attribute driving preferential attachment, where

% c=1: degree

% c=2: clustering coefficient

% c=3: betweenness centrality

% c=4: closeness centrality

% c=5: eigenvector centrality

%

% OUTPUTS

% ADJ: the adjacency matrix of the generated fitness model.

%

% Developed by Dimitrios Tsiotas, Ph.D., 15 May 2020.

tic

ADJ=zeros(n);

nodes=[1:n]';

% Create randomly the first connection

eo1=randi([1,n]); %chooses randomly an integer from the interval [1,n]

eo2=randi(setdiff([1,n],eo1)); % chooses randomly an integer from the interval [1,n]-e1

ADJ(eo1,eo2)=1;

% end of loop

Po=zeros(n,1)+(1/n);

% Attribute: Degree ---------------------------------------------------

if c==1

for i=1:n

for j=1:mo

P=degrees_und(ADJ);

P=P/sum(P)+Po′; % Po is uniform probability to connect and P/sum(P) is additional preferential probability

P=P/sum(P);

e1=find(rand<cumsum(P),1,'first'); % preferentially chooses an integer from the interval [1,n]

P([e1])=[]; % removes previous node from the selection

e2=find(rand<cumsum(P),1,'first');

ADJ(e1,e2)=1;

end

end

end

% Attribute: Degree END------------------------------------------------

% Attribute: Clustering -----------------------------------------------

if c==2

for i=1:n

for j=1:mo

P=clustering_coef_bu(ADJ);

P=P/sum(P);P(isnan(P))=0;

P=P+Po; % Po is uniform probability to connect and P is additional preferential probability

P=P/sum(P);

e1=find(rand<cumsum(P),1,'first'); % preferentially chooses an integer from the interval [1,n]

P([e1])=[]; % removes previous node from the selection

e2=find(rand<cumsum(P),1,'first');

ADJ(e1,e2)=1;

end

end

end

% Attribute: Clustering END--------------------------------------------

% Attribute: Betweenness-----------------------------------------------

if c==3

for i=1:n

for j=1:mo

P=betweenness_bin(ADJ);

P=P/sum(P);P(isnan(P))=0;

P=P+Po; % Po is uniform probability to connect and P is additional preferential probability

P=P/sum(P);

e1=find(rand<cumsum(P),1,'first'); % preferentially chooses an integer from the interval [1,n]

P([e1])=[]; % removes previous node from the selection

e2=find(rand<cumsum(P),1,'first');

ADJ(e1,e2)=1;

end

end

end

% Attribute: Betweenness END-------------------------------------------

% Attribute: Closeness-------------------------------------------------

if c==4

for i=1:n

for j=1:mo

P=closeness_und_bin(ADJ);

P=P/sum(P);P(isnan(P))=0;

P=P+Po; % Po is uniform probability to connect and P is additional preferential probability

P=P/sum(P);

e1=find(rand<cumsum(P),1,'first'); % preferentially chooses an integer from the interval [1,n]

P([e1])=[]; % removes previous node from the selection

e2=find(rand<cumsum(P),1,'first');

ADJ(e1,e2)=1;

end

end

end

% Attribute: Closeness END---------------------------------------------

% Attribute: Eigenvector-----------------------------------------------

if c==5

for i=1:n

for j=1:mo

P=eigenvector_centrality_und(ADJ);

P=P/sum(P);P(isnan(P))=0;

P=P+Po; % Po is uniform probability to connect and P is additional preferential probability

P=P/sum(P);

e1=find(rand<cumsum(P),1,'first'); % preferentially chooses an integer from the interval [1,n]

P([e1])=[]; % removes previous node from the selection

e2=find(rand<cumsum(P),1,'first');

ADJ(e1,e2)=1;

end

end

end

% Attribute: Eigenvector END-------------------------------------------

toc

end

A_3_. Null-models

The models generated with time-dynamic topological fitness under the control-attributes of degree (*k*), clustering coefficient (*C*), betweenness centrality (*CB*), closeness centrality (*CC*), and eigenvector centrality (*CE*) are shown in the following Table A1.

**Table A1:** Models generated with time-dynamic topological fitness^*^.

	*p*=	Null-model family				
		*G*_*p*_(*k*)	*G*_*p*_(*C*)	*G*_*p*_(*CB*)	*G*_*p*_(*CC*)	*G*_*p*_(*CE*)
		(degree-controlled)	(clustering-controlled)	(betweenness-controlled)	(closeness-controlled)	(eigenvector-controlled)
**Null-model ranking (*****p*****)**	1	*G*_1_(50,44)	*G*_1_(50,45)	*G*_1_(50,32)	*G*_1_(50,50)	*G*_1_(50,38)
	2	*G*_2_(100,94)	*G*_2_(100,97)	*G*_2_(100,73)	*G*_2_(100,99)	*G*_2_(100,74)
	3	*G*_3_(150,140)	*G*_3_(150,147)	*G*_3_(150,106)	*G*_3_(150,149)	*G*_3_(150,107)
	4	*G*_4_(200,184)	*G*_4_(200,195)	*G*_4_(200,163)	*G*_4_(200,199)	*G*_4_(200,146)
	5	*G*_5_(250,245)	*G*_5_(250,191)	*G*_5_(250,192)	*G*_5_(250,251)	*G*_5_(250,191)
	6	*G*_6_(300,278)	*G*_6_(300,278)	*G*_6_(300,230)	*G*_6_(300,297)	*G*_6_(300,223)
	7	*G*_7_(350,343)	*G*_7_(350,276)	*G*_7_(350,294)	*G*_7_(350,348)	*G*_7_(350,261)
	8	*G*_8_(400,384)	*G*_8_(400,374)	*G*_8_(400,313)	*G*_8_(400,397)	*G*_8_(400,278)
	9	*G*_9_(450,440)	*G*_9_(450,376)	*G*_9_(450,349)	*G*_9_(450,449)	*G*_9_(450,340)
	10	*G*_10_(500,492)	*G*_10_(500,464)	*G*_10_(500,365)	*G*_10_(500,499)	*G*_10_(500,362)
	11	*G*_11_(550,539)	*G*_11_(550,522)	*G*_11_(550,471)	*G*_11_(550,548)	*G*_11_(550,411)
	12	*G*_12_(600,588)	*G*_12_(600,595)	*G*_12_(600,404)	*G*_12_(600,598)	*G*_12_(600,441)
	13	*G*_13_(650,636)	*G*_13_(650,593)	*G*_13_(650,510)	*G*_13_(650,645)	*G*_13_(650,461)
	14	*G*_14_(700,689)	*G*_14_(700,696)	*G*_14_(700,582)	*G*_14_(700,695)	*G*_14_(700,514)
	15	*G*_15_(750,732)	*G*_15_(750,704)	*G*_15_(750,596)	*G*_15_(750,747)	*G*_15_(750,553)
	16	*G*_16_(800,788)	*G*_16_(800,735)	*G*_16_(800,643)	*G*_16_(800,794)	*G*_16_(800,575)
	17	*G*_17_(850,828)	*G*_17_(850,825)	*G*_17_(850,669)	*G*_17_(850,851)	*G*_17_(850,606)
	18	*G*_18_(900,890)	*G*_18_(900,809)	*G*_18_(900,738)	*G*_18_(900,899)	*G*_18_(900,643)
	19	*G*_19_(950,935)	*G*_19_(950,903)	*G*_19_(950,742)	*G*_19_(950,948)	*G*_19_(950,702)
	20	*G*_20_(1000,986)	*G*_20_(1000,951)	*G*_20_(1000,789)	*G*_20_(1000,999)	*G*_20_(1000,933)
	21	*G*_21_(1050,1035)	*G*_21_(1050,1011)	*G*_21_(1050,791)	*G*_21_(1050,1048)	*G*_21_(1050,775)
	22	*G*_22_(1100,1088)	*G*_22_(1100,1006)	*G*_22_(1100,859)	*G*_22_(1100,1099)	*G*_22_(1100,886)
	23	*G*_23_(1150,1132)	*G*_23_(1150,1073)	*G*_23_(1150,885)	*G*_23_(1150,1149)	*G*_23_(1150,844)
	24	*G*_24_(1200,1191)	*G*_24_(1200,993)	*G*_24_(1200,977)	*G*_24_(1200,1196)	*G*_24_(1200,873)
	25	*G*_25_(1250,1229)	*G*_25_(1250,1248)	*G*_25_(1250,993)	*G*_25_(1250,1248)	*G*_25_(1250,1170)
	26	*G*_26_(1300,1281)	*G*_26_(1300,1226)	*G*_26_(1300,977)	*G*_26_(1300,1296)	*G*_26_(1300,1000)
	27	*G*_27_(1350,1325)	*G*_27_(1350,1323)	*G*_27_(1350,1045)	*G*_27_(1350,1349)	*G*_27_(1350,1246)
	28	*G*_28_(1400,1373)	*G*_28_(1400,1368)	*G*_28_(1400,1061)	*G*_28_(1400,1400)	*G*_28_(1400,1237)
	29	*G*_29_(1450,1437)	*G*_29_(1450,1450)	*G*_29_(1450,1186)	*G*_29_(1450,1448)	*G*_29_(1450,1131)
	30	*G*_30_(1500,1475)	*G*_30_(1500,1332)	*G*_30_(1500,1219)	*G*_30_(1500,1497)	*G*_30_(1500,1135)

*Under the control-attributes of degree (k), clustering coefficient (C), betweenness centrality (CB), closeness centrality (CC), and eigenvector centrality (CE)
